# Nonsyndromic Bilateral Posterior Maxillary Supernumerary Teeth: A Report of Two Cases and Review

**DOI:** 10.1155/2018/5014179

**Published:** 2018-04-11

**Authors:** Ravi Kumar Mahto, Shantanu Dixit, Dashrath Kafle, Aradhana Agarwal, Michael Bornstein, Sanad Dulal

**Affiliations:** ^1^Department of Orthodontics, Dhulikhel Hospital, Kathmandu University School of Medical Sciences, Dhulikhel, Nepal; ^2^Department of Oral Medicine and Radiology, Dhulikhel Hospital, Kathmandu University School of Medical Sciences, Dhulikhel, Nepal; ^3^Oral and Maxillofacial Radiology, Applied Oral Sciences, Faculty of Dentistry, University of Hong Kong, Pokfulam, Hong Kong; ^4^Department of Oral and Maxillofacial Surgery, Dhulikhel Hospital, Kathmandu University School of Medical Sciences, Dhulikhel, Nepal

## Abstract

Supernumerary tooth/hyperdontia is defined as those teeth which are present in excess of the usual distribution of twenty deciduous and thirty-two permanent teeth. It can be seen in both syndromic and nonsyndromic patients. In Nepalese population, prevalence of supernumerary tooth is documented to be 1.6%. To the best of our knowledge, no studies from Nepal have reported the incidence of bilateral maxillary paramolars or the combination of unilateral maxillary paramolar and distomolar till date. Hence, we are reporting these two cases with a brief review of literature to put emphasis on incidence, prevalence, proposed hypothesis for etiology, and management of supernumerary teeth.

## 1. Introduction

Supernumerary tooth (ST) is defined as a tooth or a structure resembling tooth which forms from dental lamina in addition to the normal dental formula [1, 2]. It can occur both in the maxillae and/or mandible, unilaterally or bilaterally, solitary or in multiples, and erupted or unerupted. It can be seen in both syndromic and nonsyndromic patients. Previous researches had documented the prevalence rate of ST to be 0.2%–0.8% and 0.5%–5.3% in deciduous and permanent dentition, respectively. The male-to-female ratio for the incidence of ST was reported to range in between 1.18 : 1 and 1.5 : 1. Supernumerary teeth are also associated with larger than average teeth which reflect their multifactorial etiology. Various hypothesis were postulated by different authors to explain the phenomena of ST development, but the exact etiology is still unknown [3]. However, Brook [4] had hypothesized an interaction of environmental and genetic factors.

ST can be classified on the basis of the morphology (conical, tuberculate, supplemental, and odontomes), location (mesiodens, paramolar, distomolar, and parapremolar), position (buccal, palatal, and transverse), and orientation (vertical or normal, inverted, transverse, or horizontal). Mesiodens is the most prevalent supernumerary teeth which is seen in premaxilla. ST in the molar region is comparatively very rare [3]. Also, a very few cases have been reported about the bilateral presence of ST in the molar region [5].

Hence, we are reporting two cases of bilateral ST in the molar region. Our first case is of bilateral maxillary paramolars, whereas the other case is a combination of unilateral maxillary paramolar and distomolar. In addition, we have reviewed the existing literature to focus on incidence, prevalence, proposed hypothesis for etiology, and management of supernumerary teeth.

## 2. Case Report 1

**Figure 1:**
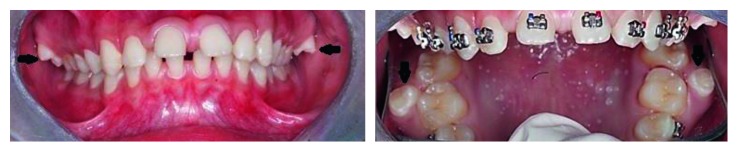
Intraoral images of Case 1 depicting bilateral maxillary paramolars (shown by arrows).

**Figure 2:**
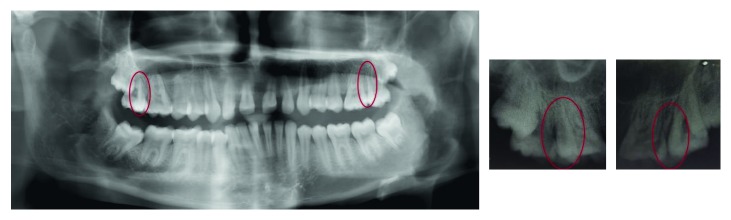
Panoramic and intraoral radiographs showing bilateral maxillary paramolars (encircled).

**Figure 3:**
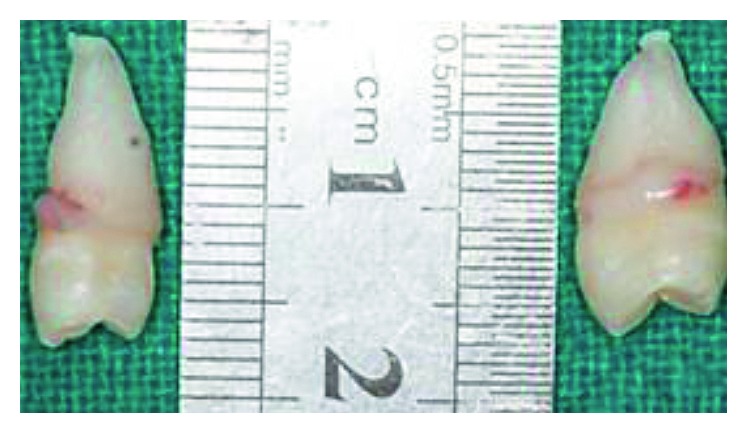
Extracted paramolars resembling maxillary premolars.

A 17-year-old male patient visited to the department of orthodontics and dentofacial orthopedics with a chief complaint of malalignment of teeth. His medical and family histories were not significant. On intraoral examination, buccally placed bilateral paramolars were present in between first and second maxillary molars (Figure 1). No clinical complications were present secondary to paramolars. Radiological investigations (intraoral periapical radiographs and panoramic radiograph) were advised to determine the root orientation (Figure 2). Both the paramolars were vertically oriented. Extractions were advised for both the paramolars to prevent any interruption in the orthodontic treatment. Extracted paramolars showed supplemental shape and form with well-defined transverse and marginal ridges resembling maxillary premolars (Figure 3). It was followed by initiation of the orthodontic treatment.

## 3. Case Report 2

**Figure 4:**
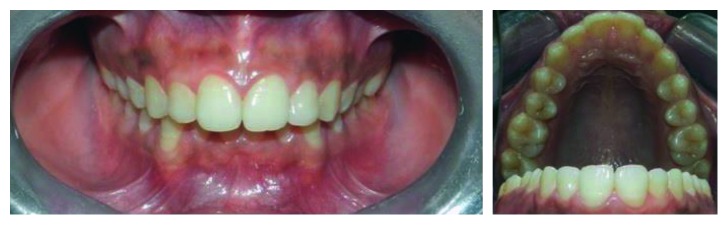
Intraoral images of Case 2.

**Figure 5:**
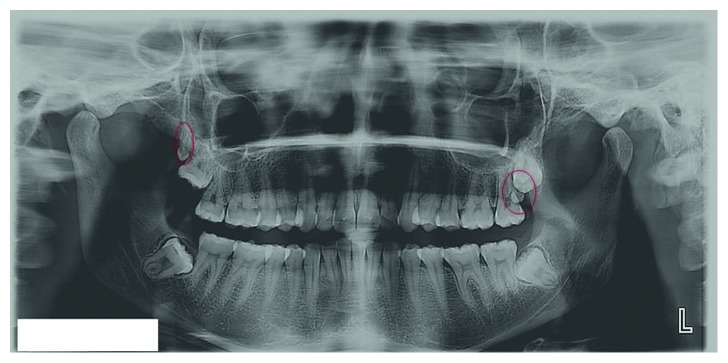
Panoramic radiograph showing maxillary the right distomolar and left paramolar (encircled).

A 23-year-old female patient visited to the department of orthodontics and dentofacial orthopedics with a chief complaint of forwardly placed upper front teeth. No significant medical and family histories were reported. On intraoral examination, fourteen teeth were present in maxillary arch (Figure 4). Clinically, maxillary third molars were missing bilaterally. She was advised for routine radiological investigations required for the orthodontic treatment. Panoramic radiograph revealed presence of a distomolar on the right side and a paramolar between left second and third molars (Figure 5). Computed tomographic scan was advised to know the accurate orientation of these impacted supernumerary teeth to formulate the treatment plan. It revealed the vertical orientation of both the impacted supernumerary teeth. Extraction of supernumerary teeth followed by the orthodontic treatment was advised to the patient.

## 4. Discussion

ST or hyperdontia as defined earlier are those teeth which are present in excess of the usual distribution of twenty deciduous and thirty-two permanent teeth [6]. Singh et al. had reported the prevalence of ST in Nepalese population to be 1.6%, which was in accordance with Hungarian (1.53%), Swedish (1.6%), and Brazilian (1.7%) population. The same study had showed the male predilection for ST with male: female ratio of 1.3 : 1 which was similar to Hungarian (1.4 : 1), British (1.4 : 1), and Brazilian (1.45 : 1) population [7–11]. Similarly, this study had also documented the prevalence of the single ST to be the most commonest (82.60%) followed by paired (15.21%) and triple ones (2.17%). Maxillary arch (98.8%) with the anterior medial region (mesiodens) and conical form was found to be the most common location and form of the supernumerary teeth in this study [7].

To the best of our knowledge, no studies from Nepal have reported the incidence of bilateral maxillary paramolars or the combination of unilateral maxillary paramolar and distomolar till date. The documented incidences similar to our cases reported in other population are briefed in Tables 1 and 2 [12, 13]. Hou et al. [14], Dhull et al. [15], Shetty [16], and Sulabha and Sameer [17] had reported the presence of bilateral maxillary paramolars similar to our first case report. Nirmala and Tirupathi [12] had documented the combination of unilateral maxillary paramolar and distomolar similar to our second case report.

The exact etiology of occurrence of ST is not known. Numerous theories have been postulated to understand their existence along with the normal dentition. Atavism theory stated the occurrence of supernumerary teeth as the phylogenetic reversion to the extinct ancestral human dentition [33]. Dichotomy theory suggested that a developing tooth bud can divide into two teeth, giving rise to ST and a normal tooth [34]. Dental lamina hyperactivity theory, the most accepted one, suggests the localized and independent hyperactivity of the dental lamina to be the cause for the development of ST [7, 35]. Niswander and Sujaku [36] also proposed the presence of an autosomal recessive gene which explains the familial tendency to ST. It have been reported in patients with syndromes like cleft lip and palate, cleidocranial dysplasia, Ehlers–Danlos syndrome type III, Fabry–Anderson's syndrome, Ellis–van Creveld syndrome, Gardner's syndrome, Goldenhar syndrome, Hallermann–Streiff syndrome, orofaciodigital syndrome type I, incontinentia pigmenti, Marfan syndrome, Nance–Horan syndrome, and trichorhinophalangeal syndrome 1 [12].

ST may be associated with different clinical complications. These can result into clinical problems like midline diastema; crowding; malocclusion due to insufficient space; dilaceration, delayed, or failure of eruption of permanent teeth; root resorption of adjacent teeth; cyst formation; cheek bite; periodontal problems; dental caries, and other difficulties related to ectopic position. These complications occur rarely, but earlier diagnosis can help to prevent these complications [4, 13].

Radiographic screening plays a significant role in identification and localization of ST, especially when they are impacted or need surgical intervention. Two-dimensional imaging modalities (periapical radiographs, occlusal radiographs, and orthopantomographs) do provide sufficient information to the clinicians, but accurate position of buccally or lingually placed ST is difficult to determine due to the superimposition by the surrounding structures [4, 13, 37]. Clark and Richards had suggested horizontal and vertical tube shift technique, respectively, to determine exact location of ST using conventional radiography. Both of these are widely accepted due to their simplicity [4, 38, 39]. Recently, Toureno et al. proposed a guideline to use three-dimensional imaging modalities (cone beam computerized tomography) along with two-dimensional imaging modalities for better assessment of ST, planning surgical intervention with minimal treatment errors [40].

There are two different school of thoughts about the management of ST. Some authors recommended the removal of ST as soon as detected, whereas others emphasized the periodic monitoring and removal only in the case of any associated pathology or hindrance to any dental treatment especially the orthodontic treatment [41–43]. Hogstrom and Andersson also suggested two different options for ST removal. According to them, ST either should be removed as early as it is identified or after completion of the adjacent tooth's root formation. However, former option could result into creation of dental phobia in young children and can disturb the growth of adjacent teeth [44]. Recently, Omer et al. suggested the optimal time for the removal of ST during 6 to 7 years, based upon their retrospective analysis. According to them, during this age interval, ST removal can be done with minimal disturbances to the adjacent teeth [1].

## 5. Conclusion

Supernumerary teeth are uncommon and generally present without causing any complications like our cases. Our cases required surgical intervention for future orthodontic treatment and planning. Although complications are rare, clinicians should be aware of early identification, proper management, and associated complications with the same.

## Figures and Tables

**Table 1:** Reported cases of paramolars.

Arch/side	Unilateral				Bilateral			
	Author	Year	Population	Location	Author	Year	Population	Location
Maxillae	Puri et al. [18]	2013	Indian	Bucally placed between second and third molars	Sulabha and Sameer et al. [17]	2015	Indian	Buccally placed between first and second molars
	Nayak et al. [19]	2012	Indian	Palatally placed between left first and second molars	Dhull et al. [15]	2012	Indian	Between first and second molars
	Nagaveni et al. [13]	2010	Indian	Buccally placed between right first and second molars	Shetty et al. [16]	2012	Indian	Palatally placed between first and second molars
					Hou et al. [14]	1995	Taiwanese	Buccally placed between first and second molars
Mandible	Ghogre and Gurav [20]	2014	Indian	Fused with the second molar	Dhull et al. [15]	2014	Indian	Mesial and lingual to the second molar
	Venugopal et al. [21]	2013	Indian	Fused with the right second molar	Nunes et al. [22]	2002	Brazil	Fused with the second molar
	Rudagi et al. [23]	2012	Indian	Fused with the left second molar				
	Salem et al. [24]	2010	Iran	Fused with the left second molar				
	Rosa et al. [25]	2010	Brazil	Fused with the right first molar				
	Ballal et al. [26]	2007	Indian	Fused with the second molar				
	Ghoddusi et al. [27]	2006	Iran	Fused with the left second molar				
	Dubuk et al. [28]	1996	Japanese	Mesial to the right second molar				
	Kumasaka et al. [29]	1988	Japanese	Two impacted paramolar on the right side				

**Table 2:** Reported cases of combination of paramolar and distomolar/bilateral paramolars.

Arch	Author	Year	Population	Location
Maxillae	Present case	2017	Nepalese	Buccally placed bilateral paramolars in between first and second molars; combination of a distomolar on the right side and a paramolar between left second and third molars
	Nirmala and Tirupathi [12]	2015	Indian	Combination of developing unerupted paramolar on the right side and distomolar on the left side
	Omal et al. [30]	2011	Indian	Bilateral paramolar between second and third molars; bilaterally impacted distomolar
	Mayfield and Casamassimo [31]	1990	Hispanic	Bilateral paramolars and distomolars
Mandible	Reddy et al. [32]	2013	Indian	Bilateral paramolar between first and second molars; bilateral distomolar with impacted second molar
